# Manganese-induced Precocious Puberty Alters Mammary Epithelial Cell Proliferation in Female Rats

**DOI:** 10.1210/endocr/bqaf052

**Published:** 2025-03-19

**Authors:** Alina M Hamilton, Vinod K Srivastava, Jill K Hiney, William L Dees, Robert K Dearth

**Affiliations:** School of Integrative Biological and Chemical Sciences, College of Sciences, University of Texas Rio Grande Valley, Edinburg, TX 78539, USA; Lineberger Comprehensive Cancer Center, School of Medicine, University of North Carolina at Chapel Hill, Chapel Hill, NC 27599, USA; Center for Human Health and the Environment, North Carolina State University, Raleigh, NC 27606, USA (Current); Department of Veterinary Integrative Biosciences, College of Veterinary Medicine, Texas A&M University, College Station, TX 77843-4458, USA; Department of Veterinary Integrative Biosciences, College of Veterinary Medicine, Texas A&M University, College Station, TX 77843-4458, USA; Department of Veterinary Integrative Biosciences, College of Veterinary Medicine, Texas A&M University, College Station, TX 77843-4458, USA; School of Integrative Biological and Chemical Sciences, College of Sciences, University of Texas Rio Grande Valley, Edinburg, TX 78539, USA

**Keywords:** precocious puberty, breast cancer risk factors, hormone-regulated proliferation, autocrine proliferation, manganese, early puberty

## Abstract

Precocious puberty (PP) is an established breast cancer risk factor. In the normal mammary gland, hormone receptor-positive (HR+) cells rarely proliferate. In breast cancer, proliferating epithelial cells are often HR+. It is not known if PP can modify this population of proliferating HR+ cells. Previously, we established a manganese-induced precocious puberty (MnPP) model to study the effects of PP on mammary gland development in female rats. Here, we characterized the distribution of HR+ proliferating mammary epithelial cells in prepubertal and adult rodents, in association with precocious puberty. Female rats were exposed daily to 10 mg/kg manganese chloride or saline (control) from postnatal day (PND) 12 to PND 30. Mammary glands were collected on PNDs 30 and 120, processed for western blot analysis and double immunofluorescence staining for proliferating cell nuclear antigen and progesterone receptor or estrogen receptor. MnPP increased the percentage of HR+ mammary epithelial cells coexpressing proliferating cell nuclear antigen relative to normally developed controls at PND 30. This correlated with increased expression of estrogen receptor-regulated proteins in MnPP mammary glands relative to controls at PND 30, including FOXA1, AREG, and c-Myc. Conversely, at PND 120 relative to PND 30, proliferating HR+ cells remained chronically elevated in MnPP mammary glands at PND 120, which coincided with decreased expression of cell-cycle regulator, p27, and increased expression of progesterone receptor-regulated markers, EREG and sp1. Collectively, these results suggest early puberty alters steroidal regulation of classic proliferative mechanisms in the prepubertal gland with increased prevalence of high-risk proliferating HR+ cells.

Epidemiological data have demonstrated that an earlier age at menarche is associated with a 10% to 20% increased risk for developing breast cancer (1-5). There are 2 general theories regarding the mechanism driving this association, both of which are related to the timing of endogenous estradiol (E_2_) and progesterone (P_4_) exposure. First, an earlier than normal pubertal onset might extend the number of years the mammary gland is responsive to these mitogenic ovarian steroids (3-5). In the second theory, puberty is emphasized as a critical developmental window where mammary cells are vulnerable to surging hormones, and when cellular reprogramming might have a lasting impact on breast health (6). In 2012, a large epidemiological study provided evidence that the age at hormonal exposure, rather than just the total number of menstrual cycles, may be of greater importance in breast cancer risk than previously thought (7). Supporting this, a comparison of hereditary breast cancer in groups of monozygotic twins suggests that the risk for breast cancer is due in part to exposure of the developing mammary gland to a complex milieu of hormones and growth factors normally accompanying puberty (8). Therefore, the hormones accompanying puberty, if present earlier than normal, might promote an alternative mammary epithelial cell phenotype that is more sensitive to repetitive ovarian hormone stimulation, eventually leading to uncontrolled cell growth.

During pubertal development, E_2_, via the estrogen receptor (ER), is the first and primary steroidal stimulus of mammary epithelial cell proliferation and ductal elongation (9-12). Following E_2,_ P_4_ induces alveologenesis and side branching via the progesterone receptor (PR) (13). In the normal mammary gland, hormone receptor-positive (HR+) cells rarely proliferate themselves but instead drive proliferation of adjacent cells through paracrine signaling (14-16). Specifically, hormones control the release of paracrine factors, including amphiregulin (AREG) and epiregulin (EREG), from HR+ cells to stimulate proliferation of neighboring HR-negative (HR-) cells (15, 17, 18). Interestingly, in mammary hyperplasia and ER-positive (ER+) breast cancer, a change has been observed where a large percentage of proliferating cells also express the HR’s ER and/or PR (14, 18-20). Thus, determining if early puberty can produce a similar change among HR+ cells in the post-vaginal opening (VO) mammary gland would provide insight in aberrant mammary gland development and breast cancer.

Until recently, investigating the effects of early puberty on mammary gland development and breast cancer risk has been difficult because of the lack of a strong experimental model to study this relationship. Manganese (Mn) is an essential nutrient required for normal mammalian physiological processes, including growth and reproduction (21, 22). Using a rat animal model, our group has further established a role for Mn in the central activation of GnRH secretion, the hypothalamic peptide responsible for initiating pubertal development (23-25). Using this model, we previously demonstrated that prepubertal exposure to elevated Mn resulted in precocious puberty, including accelerated E_2_-regulated mammary gland development, aberrant ER protein expression, and sustained proliferation and ductal hyperplasia in the adult mammary gland of female rats (26). In the current study, we build on our previous work using our Mn-induced precocious puberty (MnPP) model to continue our exploration of HR-related changes in the developing mammary gland. Specifically, we investigate the effect of MnPP on expression of PR-positive (PR+) epithelial cells and associated proteins in the developing and adult mammary gland, we characterize the effect of MnPP on the distribution of proliferative HR+ epithelial cells in prepubertal and adult female rats and identify cellular proteins that might be involved in this process. Finally, we test the validity of our MnPP model by exposing post-VO females to Mn and evaluating the effect on mammary gland growth and E_2_ serum levels.

## Materials and Methods

### Animals

Female Sprague Dawley rats used in this experiment were generated from our colony under controlled conditions of light (lights on: 0600 hours; lights off: 1800 hours), temperature (23 °C), and with ad libitum access to deionized water and food (Harlan Teklad 2016 rodent chow; Harlan Laboratories, Indianapolis, IN). All procedures were approved by the University Institutional Animal Care and Use Committee at both Texas A&M University and the University of Texas Rio Grande Valley and were in accordance with the National Institutes of Health Guidelines for the Care and Use of Laboratory Animals.

### MnPP Experimental Design

Sprague Dawley rats were bred and allowed to deliver their pups normally. Each litter was culled to 10 to 12 pups while maintaining 5 to 6 females in each litter. Half of the female pups from each dam were designated as saline-treated (controls), whereas the other half were designated as MnPP, as previously described (23-26). Briefly, on postnatal day (PND) 12, females were dosed daily via gastric gavage with either 10 mg/kg MnCl_2_ or an equal volume of saline. Dosing continued until PND 29 for a total of 18 days. On PND 30, half of the females in each treatment group were sacrificed and mammary glands were harvested for analysis as described later. Ninety days later, the other half in each group were sacrificed and glands harvested for analysis between PND 119 and 122 (referred to throughout as PND 120). All females collected at PND 30 were confirmed to be immature (anestrous) and all adult PND 120 females were confirmed to be in the diestrus stage of the estrous cycle. The criteria for confirming these developmental stages have been described previously along with puberty-related hormone levels that normally accompany the onset of puberty (27, 28).

### Post-VO Mn Experimental Design

In a separate experiment, female rats were checked daily for VO beginning on PND27, which marks pubertal onset. Once VO was observed, females were dosed daily via gastric gavage with either 10 mg/kg MnCl_2_ or an equal volume of saline for a total duration of 18 days (Supplemental Figure 1A) (29). Half of the females in each treatment group were sacrificed between PND 45 and 55 (referred to throughout as PND 50) via decapitation, and both trunk blood and mammary glands were harvested and prepared for further analysis as described below. The remaining females in each group remained housed under normal conditions for 90 additional days, and trunk blood and mammary glands were collected between PND 135 and PND 145 (referred to throughout as PND 140). All females were confirmed to be in the diestrus phase of the estrous cycle by observing vaginal cytology and confirming minimal intraluminal uterine fluid on the day of sacrifice.

### Cell Culture

The ER+ MCF7 human breast cancer cell line was purchased from the American Type Culture Collection (Manassas, VA, USA, RRID:CVCL_0031). MCF7 cells were maintained in DMEM (Corning Inc., Corning, NY) supplemented with 5% fetal bovine serum and 1% Pen-Strep antibiotic in a humidified environment at 37 °C and 5% CO_2_. Cells at approximately 70% confluency were seeded into 6-well dishes, transfected with an estrogen response element upstream of a luciferase reporter gene, and subjected to treatment with various concentrations of MnCl_2_, E_2_ as a positive control, or DMEM as a negative control. Stock E_2_ solutions were prepared in ethanol (EtOH) at 50 mg/mL, then diluted to 10 nM in phenol red-free DMEM. A 10-nM E_2_ treatment was used as a positive control for canonical ER stimulation because this concentration of estradiol has been proven in the stimulation of MCF7 cells (30). MnCl_2_ treatments were prepared in phenol red-free DMEM. Because of the potential estrogenic properties of EtOH in vitro (31), EtOH was not included in the negative control or MnCl_2_ treatment groups to maintain nonestrogenic conditions. The concentrations of Mn chosen for this experiment were based on a dose conversion of normal human dietary Mn intake within estimated safe and adequate dietary intake ranges as reported by the Agency for Toxic Substances and Disease Registry, 2012 (32). All Mn doses were based on the 3% to 5% Mn absorption rate for a 70-kg individual and are represented in Supplemental Table 1 (29).

### Transfection and Luciferase Reporter Assay

The day before transfection, MCF7 cells were seeded at 500 000 cells per well in 6-well dishes in complete medium. Three hours before transfection, the cells were washed with PBS and cultured in serum-free media without antibiotics. The cells were then co-transfected with 250 ng pGL2 3X-ERE-TATA-luc and 100 ng of pGLK in Opti-MEM using Lipofectamine 2000 (Invitrogen, Carlsbad, CA) for 24 hours according to the manufacturer's instructions. The cells were then treated and prepared for luciferase reporter assays as described later. The pGL2 3X-ERE-TATA-luc plasmid that contains the luciferase reporter gene under the control of 3 estrogen response elements was a gift from Donald McDonnell (Addgene plasmid #11354) (33).

Twenty-four hours after transfection, the transiently transfected cell line, MCF7-3X-ERE-TATA-luc, was washed with Dulbecco's PBS and media was changed to estrogen-free conditions using phenol red-free DMEM containing 5% dextran-coated charcoal-stripped fetal bovine serum for 3 hours. Cells were then treated in triplicate with either 10 nM E_2_ (positive control), DMEM (negative control), 10 nM MnCl_2_, 50 nM MnCl_2_, 100 nM MnCl_2_, 250 nM MnCl_2_, or 1 μM MnCl_2_ prepared in estrogen-free media for 24 hours.

Following treatment, luciferase reporter assays were carried out using Promega Dual Luciferase Reporter Assay Kit (Promega, Madison, WI) according to the manufacturer's instructions with the following modifications. Cells were washed once with PBS before adding 250 μL of chilled passive lysis buffer to each well. Cells were then lysed with a cell scraper, pipetted up and down briefly, and taken through 2 rapid freeze-thaw cycles. A total of 20 μL of each cell lysate was then transferred to a clean tube containing 100 μL of LAR II reagent and mixed carefully by pipetting. Firefly reading was immediately taken using the 20/20n luminometer (Turner Biosystems, Sunnyvale, CA) and recorded. Immediately after reading, 100 μL of Stop and Glo reagent was added to the sample, vortexed briefly, and Renilla reading was quickly taken and recorded. Luciferase activity is expressed as the Firefly to Renilla ratio.

### Immunohistochemistry (IHC)

Sample preparation for histological and IHC analysis was conducted as we have described previously with minor modifications (26, 34). Abdominal mammary gland 4 was collected, placed in cassettes, fixed overnight in 4% paraformaldehyde in PBS. The following day, samples were sent to the Histology Core at Texas A&M University for paraffin embedding. Multiple serial sections of each mammary gland were obtained at a thickness of 5 μm, placed on slides, deparaffinized using xylene, and then gradually rehydrated using a series of graded ethanol. Following rehydration, antigen retrieval was achieved by incubation of tissue sections for 20 minutes at 100 °C in sodium citrate buffer (10 mM Sodium citrate, 0.05% Tween 20, pH 6.00), using a water bath to maintain constant temperature. Endogenous peroxidase activity was blocked by a 10-minute incubation in 3% H_2_O_2_ diluted in methanol, blocked for 1 hour at room temperature (RT) in normal goat serum using the Vector ABC Elite Kit (Vector Laboratories, Burlingame, CA), and incubated overnight with either anti-ERα (1:100, Abcam Cat# ab16660, RRID:AB_443420) or anti-PR (1:500, Abcam Cat# ab16661, RRID:AB_443421) primary antibody at 4 °C. Slides were then incubated in biotinylated goat anti-rabbit secondary antibody (Vector Laboratories) for 1 hour at RT, washed 3× for 10 minutes in PBS, and incubated in avidin peroxidase for 30 minutes using the Vector ABC Elite Kit. The chromogenic reaction was carried out by a short incubation in 3′-3′diaminobenzidine solution (Vector Laboratories). All slides were then washed with water, counterstained in hematoxylin (Fisher Scientific, Hampton, NH), dehydrated with graded ethanol, cleared with xylenes overnight, and coverslipped with permount. All immunohistochemistry (IHC) images were taken with the Leica ICC50 microscope and camera. Analysis of ER and PR expression at PND 120 was performed as previously described (26). Images of ERα and PR protein expression patterns adjacent to human invasive lobular breast carcinoma and ductal carcinoma in situ were provided by The Human Protein Atlas (available at http://www.proteinatlas.org) (35).

### Double Immunofluorescence

For immunofluorescence (IF) analysis, 2 to 4 mammary gland tissue sections per animal were obtained and processed as described previously, with the following modifications. Following rehydration, antigen retrieval was performed by incubating tissue sections in Tris-EDTA buffer (10 mM Tris base, 1 mM EDTA solution, 0.05% Tween 20, pH 9.00) for 20 minutes at 100 °C. Slides were then incubated for 10 minutes in 3% H_2_O_2_ diluted in methanol to reduce autofluorescence of red blood cells and blocked for 1 hour in normal goat serum using the Vector ABC Elite Kit (Vector Laboratories). Following blocking, tissue sections were incubated overnight at 4 °C with anti-PCNA primary antibody (1:200, Abcam Cat# ab29, RRID:AB_303394) followed by incubation with Alexa Fluor 488 goat anti-mouse secondary antibody (1:1000; Thermo Fisher Scientific Cat# A28175SAMPLE, RRID:AB_2610666) for 1 hour at RT. Next, IF slides were washed 3× for 10 minutes in PBS and incubated overnight at 4 °C with either anti-ERα (1:200, (Millipore Cat# 06-935, RRID:AB_310305) or anti-PR (1:200, Abcam Cat# ab16661, RRID:AB_443421) primary antibodies. Slides were then incubated with Alexa Fluor 594 goat anti-rabbit secondary antibody (1:1000; Thermo Fisher Scientific Cat# A78956, RRID:AB_2925779) for 1 hour at RT, counterstained for 6 minutes in 2.86 μM DAPI, and cover slipped with Diamond Superfade Mountant (ThermoFisher, Waltham, MA). A negative control (no primary antibody) slide consisting of mammary gland tissue was included in each batch for assessment of secondary antibody nonspecific binding. All washes were performed with PBS.

For each treatment condition and time point, we determined the percentage of mammary epithelial cells positive for ER, PCNA, ER+ PCNA, PR, or PR+ PCNA. For each animal, 3 randomly selected 4-mm (2) regions of interest were selected from each tissue section for analysis. Each region of interest was imaged in its entirety, resulting in 16 to 24 40× images for each gland. Approximately 2700 cells were manually counted and analyzed for each gland. The number of single-positive and double-positive cell nuclei relative to the total number of mammary epithelial nuclei (Dapi) was averaged per animal and then compared between treatment groups. Double IF assays were conducted in 2 separate assays: (1) ER+ PCNA and (2) PR+ PCNA. This resulted in 2 individual measures of PCNA (ie, PCNA 1 and PCNA 2) for each treatment group on separate tissue sections. In a validation experiment to ensure intratissue heterogeneity did not alter results, we compared PCNA quantification across these 2 multiplex assays for the same animals and found high agreement between assays, with nonsignificant difference in means within treatment groups (*P* = .14). All images were taken with the Fluoview FV10i Confocal Microscope (Olympus, Center Valley, PA, USA).

### Mammary Epithelial Cell Isolation and Western Blot

Enriched epithelial cell lysates were isolated following methods previously described (36). On PND 30 and 120, mammary glands 3 (caudal thoracic), 4 (abdominal), and 5 (cranial inguinal) per female were harvested, lymph nodes removed and immediately placed in 10 mL of chilled mammary epithelial cell digestion buffer containing 100 U/mL hyaluronidase, 2 mg/mL Collagenase A in Ham's DMEM/F12 medium (Corning Inc., Corning, NY). The glands were diced in solution for 2 minutes and digested for 1 to 2 hours at 37 °C with shaking at 180 rpm until dissolved. After digestion, the cell suspension was brought up to 35 mL with ice-cold F12 media and centrifuged at 800 rpm for 3 minutes at 4 °C. The first pellet was saved, whereas the supernatant was transferred and centrifuged for 10 more minutes at 1500 rpm to form a second pellet. The first and second pellets were combined, washed 3× by centrifuging in 35 mL of cold Ham's F12 for 5 minutes at 1000 rpm, and supernatant discarded. Isolated mammary epithelial cell fractions were homogenized on ice in 1% Igepal CA-630, 20 mM Tris-HCl, pH (8.0), 137 mM NaCl, 2 mM EDTA, 10% glycerol, 10 mM sodium fluoride, 1 mM sodium orthovanadate, 1 mM phenylmethylsulfonyl fluoride, and 0.25% protease inhibitor cocktail (Sigma-Aldrich, St. Louis, MO). The homogenates were incubated on ice for 30 minutes and centrifuged at 12 000*g* for 15 minutes at 4 °C. Protein concentration was determined by the BSA assay (Bio-Rad Laboratories, Hercules, CA) using BSA as a standard. Western blot analysis was performed as previously described (Dearth et al, 2014), using anti-Sp1 (1:500, Sigma-Aldrich Cat# 07-645, RRID:AB_310773), anti-p27 (1:500, (Cell Signaling Technology Cat# 3686, RRID:AB_2077850), anti-c-myc (1:500, Cell Signaling Technology Cat# 5605, RRID:AB_1903938), anti-amphiregulin (AREG) (1:750, Bioss Cat# bs-3847R, RRID:AB_10886273), anti-epiregulin (EREG) (1:500, Santa Cruz Biotechnology Cat# sc-376284, RRID:AB_11014189), and anti-HNF-3α/FoxA1 (FOXA1) (3 µg/µL, Novus Cat# NBP2-45269, RRID:AB_2687601 and anti-PR (1:500, Thermo Fisher Scientific Cat# MA1-410, RRID:AB_2164327). Goat anti-rabbit secondary antibody (1:50 000 Santa Cruz Biotechnology Cat# sc-2004, RRID:AB_631746) was used to detect Sp1, p27, c-myc, and AREG, and goat anti-mouse secondary antibody (1:50 000 Santa Cruz Biotechnology Cat# sc-2005, RRID:AB_631746) was used for EREG, FOXA1, PR, and β-actin (1:50 000; Sigma-Aldrich Cat# A1978, RRID:AB_476692). All target proteins were normalized to anti-β-actin.

### Mammary Gland Whole Mount and Morphological Analysis

Mammary gland whole mounts were processed and quantified as previously described (26, 34). Following processing, the tissues were stored indefinitely in methyl salicylate and analyzed using the Leica EZ4D microscope. Terminal ductal structures were counted in each gland and analyzed with a total of 4 mammary glands analyzed for controls and 3 glands for Mn-treated females. The mean number of each structure per group was quantified and statistical analysis between groups was carried out as described next.

### Mn Tissue Metal Analysis

Whole blood and mammary glands from both PND 50 and PND 140 females were weighed and collected in tubes that had been washed of all contaminants by soaking in a 4% nitric acid bath for a minimum of 4 hours. All tissues were subsequently lyophilized in a Labconco Freezezone 6 freeze dry system (Kansas City, MO) and dry weight was recorded. All samples were subjected to acid digestion using the US EPA Method 200.3 (37). Quality controls included water-only samples, method blanks, and spiked Mn-only samples. All samples were analyzed using the Perkin Elmer AAnalyst 800 Atomic Absorption Spectrometer (Shelton, CT). Three replicates per sample were analyzed with the addition of a 0.1% Pd and 0.06% Mg(NO_3_)_2_ matrix modifier solution: 15 μL of sample was added together with 5 μL of matrix modifier solution. A correlation coefficient of 0.998 or better was obtained for each standard curve performed in all analyses.

### Statistical and Serum Hormone Analysis

Trunk blood was collected from control and Mn-treated animals and allowed to coagulate at RT for 20 minutes before being centrifuged at 3000*g* for 30 minutes. The serum was removed, aliquoted in 100-µL volumes, and stored at −80 °C for later analysis. Serum E_2_ was determined using a mouse/rat estradiol ELISA assay (Calbiotech Cat# ES180S-100, RRID:AB_2756386) according to manufacturer's instructions. The assay sensitivity was 3 pg/mL.

All data were assessed for normality using Shapiro-Wilk normality tests, and differences between MnPP and control groups were analyzed using the 2-tailed, unpaired Student *t*-test, assuming random sampling, as well as Welch's 2-sample *t*-tests or Wilcoxon rank-sum test when data were not normally distributed. Probability values of <.05 were considered statistically significant. Analysis and data visualization were conducted using INSTAT and PRISM (GraphPad, San Diego, CA), as well as R version 4.2.2.

## Results

### Effect of MnPP on Proliferating HR+ Epithelial Cells at PND 30 and 120

**Figure 1.**
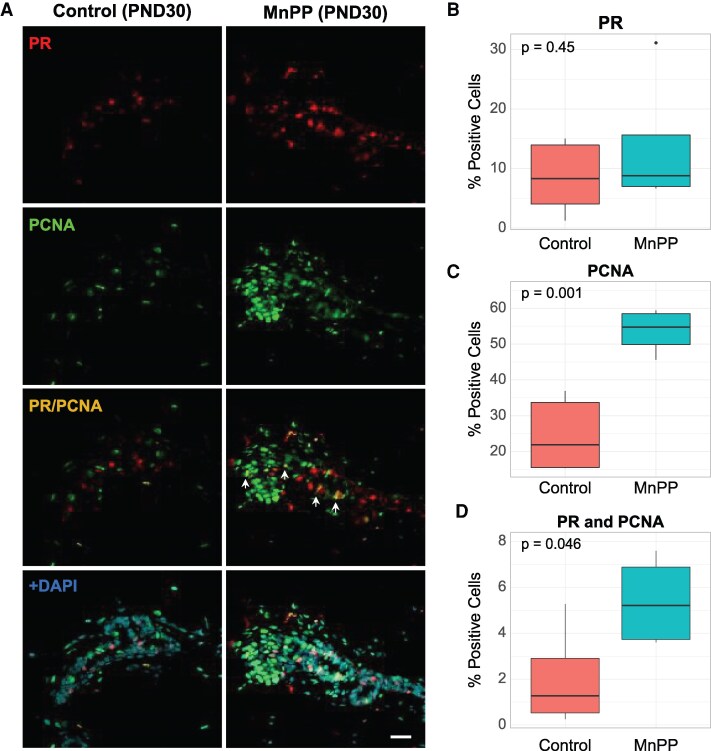
Expression of PR+ and PCNA+ cells at PND 30. Representative IF images (A) show PCNA+, PR+, and double-labeled cells expressed in mammary glands of PND 30 saline-treated and MnPP females. (B-D) Boxplots depict percentage of each cell type comprising the gland at PND 30, quantified across 2-4 serial sections from each animal. *P* values were determined using Welch's 2-sample *t*-test. All boxplots display the median, interquartile range, and minimum and maximum values for each group. White arrows indicate dual-labeled cells. Scale bar = 20 µM. Control: N = 5; MnPP: N = 4. IF, immunofluorescence; PND, postnatal day; PR, progesterone receptor.

Previously, we demonstrated that the MnPP phenotype was characterized by a prepubertal increase in serum E_2_ and accelerated prepubertal mammary epithelial cell proliferation at PND 30, which resulted in increased proliferation and ERα expression in the adult mammary gland at PND120 (26). Therefore, we assessed whether MnPP also alters prepubertal expression of PR, the receptor that drives alveologenesis in the developing mammary gland and human breast. Figure 1 depicts representative IF images (Fig. 1A) and cell counts (Fig. 1B-1D**)** of abdominal mammary glands at PND 30 from female rats treated as juveniles with either Mn (MnPP) or saline (controls). Although there was no difference in the mean percentage of PR+ cells between groups at PND 30 (Fig. 1B, Supplemental Table 2 (29)), there was a > 2-fold increase in the percentage of proliferating (PCNA+) luminal epithelial cells in glands from MnPP females at PND 30 (Fig. 1C, Supplemental Table 2 (29)). Interestingly, a significantly higher proportion of these proliferating cells coexpressed PR (PR+/PCNA+) in prepubertal glands from MnPP females (Fig. 1D, Supplemental Table 2 (29)), suggesting a shift from paracrine to autocrine mediated proliferation among a subset of HR+ mammary epithelial cells in precociously developed females.

**Figure 2.**
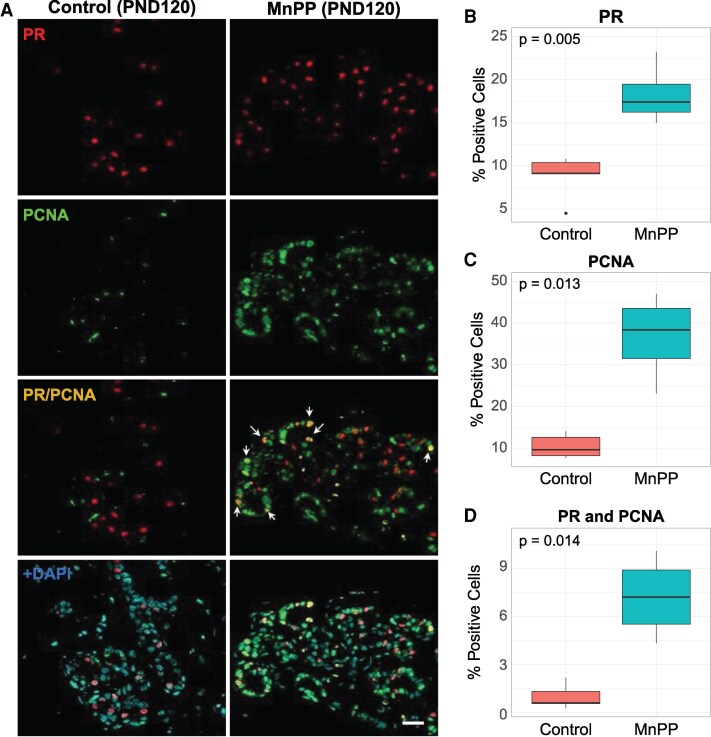
Effect of MnPP on expression of PR+ and PCNA+ cells in the adult virgin mammary gland. Representative IF images (A) show PCNA+, PR+, and double-labeled PR+/PCNA+ cells in mammary glands of PND 120 saline-treated control and MnPP females. (B-D) Boxplots depict percentage of each cell type comprising the gland at PND 120, quantified across 2-4 serial sections from each animal. *P* values were determined using Welch's 2-sample *t*-test. All boxplots display the median, interquartile range, and minimum and maximum values for each group. White arrows indicate double-labeled cells. Scale bar = 20 µM. Control N = 5; MnPP N = 4. IF, immunofluorescence; PND, postnatal day; PR, progesterone receptor.

In contrast to our findings at PND 30, we identified a significant increase in the percentage of PR+ cells in MnPP adult mammary glands relative to controls at PND 120, which is 90 days after the last dose of Mn (Fig. 2A and 2B, Supplemental Table 2 (29)). Interestingly, although the mean percentage of PR+ cells remained similar in controls between PND 30 (8.51%) and PND 120 (8.79%), there was a trending, but nonsignificant, increase in the mean percentage of PR+ epithelial cells from 13.83% at PND 30 to 18.27% at PND 120 in MnPP glands (Supplemental Table 2 (29)). Concordant with our previous work (26) and following the same trend observed at PND30 for MnPP females, the percentage of PCNA+ cells remained elevated in MnPP glands relative to controls at PND 120 (Fig. 2C, Supplemental Table 2 (29)). Similarly, the population of proliferating PR+ cells was maintained in precociously developed adult females, with an approximately 7-fold increase in the mean percentage of PR+/PCNA+ cells in MnPP females relative to controls at PND 120 (Fig. 2A and 2D, Supplemental Table 2 (29)). This was in stark contrast to the prevalence of dual labeled PR+ proliferating cells (PR+/PCNA+) in control females, which were rarely observed in control glands at any time point (PND 30: 2.04%; PND 120: 0.99%; Supplemental Table 2 (29)).

**Figure 3.**
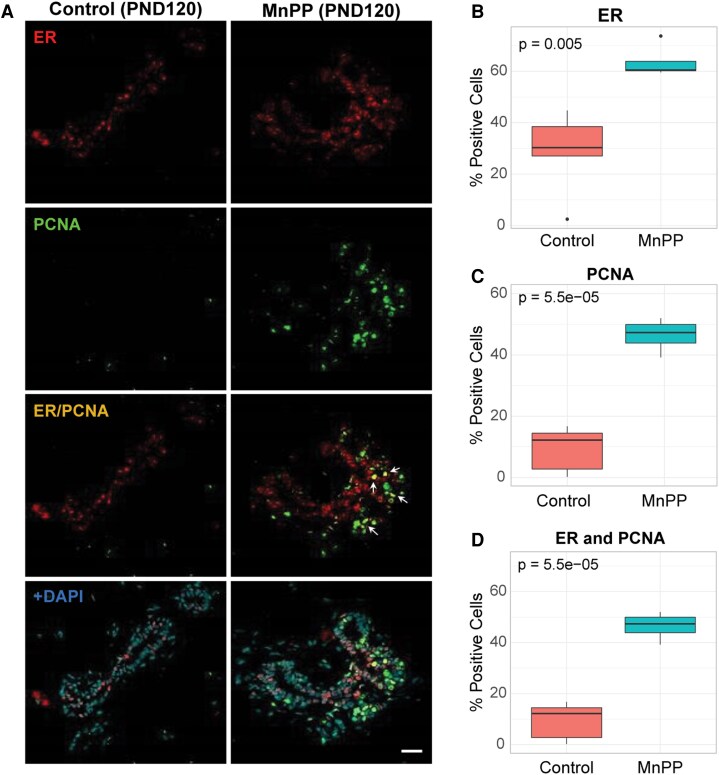
Effect of MnPP on expression of ER+ and PCNA+ cells in the adult virgin mammary gland. Representative IF images (A) show PCNA+, ER+, and dual-labeling ER+/PCNA+ cells in mammary glands of PND 120 saline-treated control and MnPP females. (B-D) Boxplots depict percentage of each cell type comprising the gland at PND 120, quantified across 2-4 serial sections from each animal. *P* values were determined using Welch's 2-sample *t*-test. All boxplots display the median, interquartile range, and minimum and maximum values for each group. White arrows indicate dual-labeled cells. Scale bar = 20 µM. Control N = 5; MnPP N = 4. ER, estrogen receptor; IF, immunofluorescence; PND, postnatal day.

Results for ER+ cells mirrored PR-related findings at PND120. The mean percentage of ER+ mammary epithelial cells was increased by >2-fold in MnPP adult mammary glands compared to controls at PND120 (Fig. 3A and 3B, Supplemental Table 2 (29)). The frequency of PCNA+ cells was also higher in MnPP females relative to controls at PND120 (Fig. 3A and 3C), displaying strong concordance in PCNA quantification between serial sections in PR/PCNA (Fig. 2) and ER/PCNA (Fig. 3) multiplex experiments (see Methods, *P* = 0.14). Finally, the mean percentage of dual-labeled ER+/PCNA+ cells was greater in MnPP mammary glands compared to controls at PND 120 (Fig. 3A and 3D, Supplemental Table 2 (29)).

### MnPP-associated Changes in PR and ER-regulated Protein Expression at PND 30 and 120

**Figure 4.**
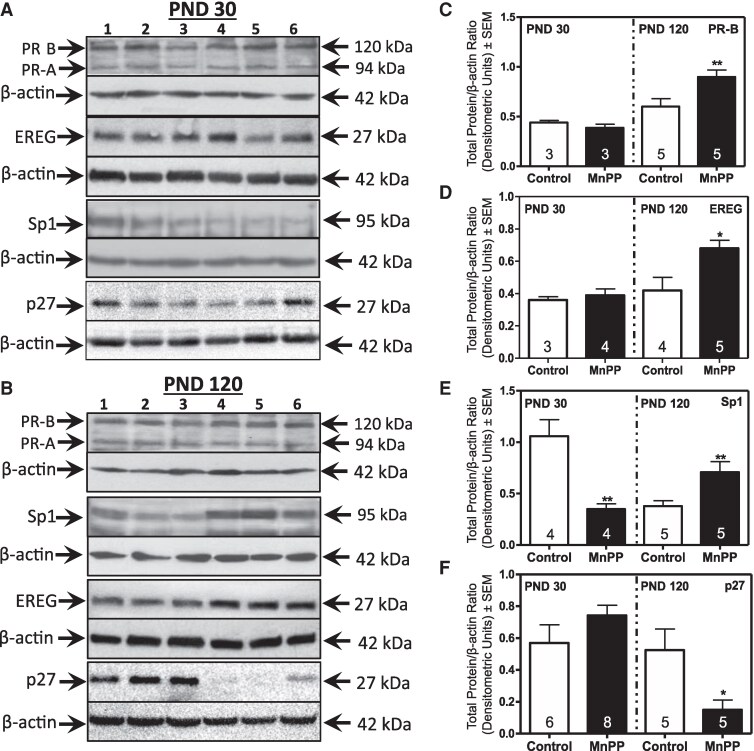
Effect of MnPP on PR-associated protein expression. Representative western blots (A and B) show expression of PR-B, EREG, P27, and SP1 in epithelial cell lysates from controls (lanes 1-3) and MnPP (lanes 4-6) animals at PND 30 and PND 120, respectively. Bar graphs show changes in the mean (±SEM) of respective protein expression levels for PR-B (C), EREG (D), Sp1 (E), and p27 (F), at PND 30 (left) and PND 120 (right). Animal numbers (N) are shown within the bars. Data was analyzed by Student *t*-test. *P < .05; **P < .01.

**Figure 5.**
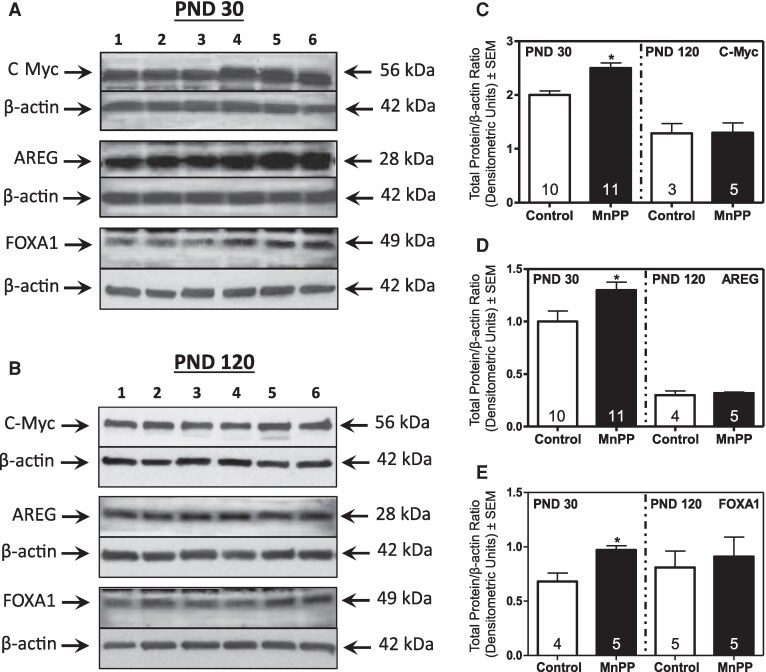
Effect of MnPP on ER-associated protein expression. Representative western blots (A and B) show the expression of c-myc, AREG, and FOXA1 in enriched epithelial cell lysates from controls (lanes 1-3) and Mn-treated (lanes 4-6) at PND 30 and PND 120, respectively. Bar graphs show changes in the mean (±SEM) of respective protein expression levels for c-Myc (C), AREG (D), and FOXA1 (E) at PND 30 (left) and PND 120 (right). Animal numbers (N) shown within the bars. Data were analyzed by Student *t*-test. *P < .05.

To understand the role of HRs in the MnPP-associated proliferative phenotype, we investigated expression of key proteins related to ER- and PR-regulated proliferation and cell-cycle control. Using western blot analysis, we observed no differences in expression of PR isoform B (PR-B) (Fig. 4A and 4C), EREG (Fig. 4A and 4D), or p27 (Fig. 4A and 4F) between MnPP and control females at PND 30, whereas Sp1 was suppressed in MnPP animals (Fig. 4A and 4E). However, by PND 120, expression of PR-associated proteins PR-B, EREG, and Sp1 were significantly elevated in glands from MnPP females relative to controls, and expression of p27 was decreased (Fig. 4B-4F). With regard to ER-regulated proteins, at PND30, MnPP resulted in increased protein expression of c-myc, AREG, and the ERα co-regulator FOXA1 (Fig. 5A and 5C-5E). Conversely, although histological analysis revealed higher frequencies of proliferating ER+ cells in glands from MnPP adult females (Fig. 3), the accompanying expression levels of key ER-associated proteins from mammary epithelial cell lysates (ie, c-myc, AREG, FOXA1) were similar to controls by PND 120 (Fig. 5B-5E). Thus, these data suggest that proliferative changes in the prepubertal gland were likely ER-driven, whereas PR-associated signaling was associated with sustained proliferation in the adult gland.

### PR and ER**α** Expression in Rat and Human Breast Tissue

**Figure 6.**
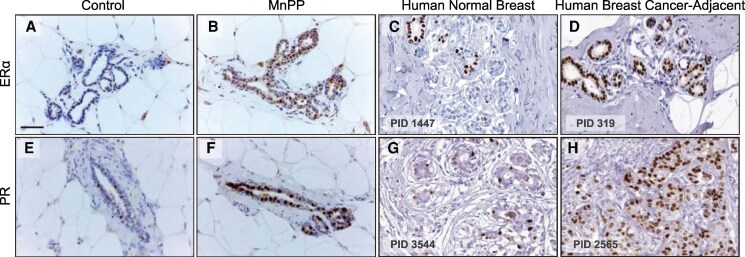
ER and PR expression in the MnPP rat and in human cancer-adjacent tissue. (A and B) Representative IHC images depict ERα expression in PND120 mammary glands from control and MnPP female rats, respectfully. (C) ERα expression in human normal breast epithelium and (D) tissue adjacent to ductal carcinoma in situ (DCIS). PR+ expression in control (E) and MnPP rat mammary glands at PND 120 (F). PR+ expression in normal breast epithelium (G) and epithelial tissue adjacent to human invasive lobular carcinoma (panel H). Images of ERα and PR protein expression patterns in human breast tissue were provided by The Human Protein Atlas (available at http://www.proteinatlas.org)^35^. Scale bar = 0.01 mm. Erα, estrogen receptor alpha; IHC, immunohistochemistry; MnPP, manganese-induced precocious puberty; PID, patient ID; PR, progesterone receptor.

The expression of PR and ERα in nonneoplastic mammary glands from MnPP adult female rats followed a similar expression pattern to that of normal human breast tissue and breast cancer-adjacent tissue from the human protein atlas (35) (Fig. 6). Figures 6A and 6B are representative IHC images illustrating elevated ERα expression in histologically normal mammary glands from precociously developed MnPP rats relative to controls at PND 120. This is compared to ERα expression in human normal breast (Fig. 6C) and breast tissue adjacent to ductal carcinoma in situ (Fig. 6D). Figures 6E and 6F show representative mammary glands stained for PR in adult control and MnPP rats at PND 120. We noted that the increased frequency of PR+ cells in MnPP females relative to controls is similar to the change in PR expression levels in breast tissue adjacent to human lobular breast carcinoma (Fig. 6H) relative to normal breast (Fig. 6G).

### Effect of Post-VO Mn Exposure on Estrogen Levels and Mammary Gland Growth

**Figure 7.**
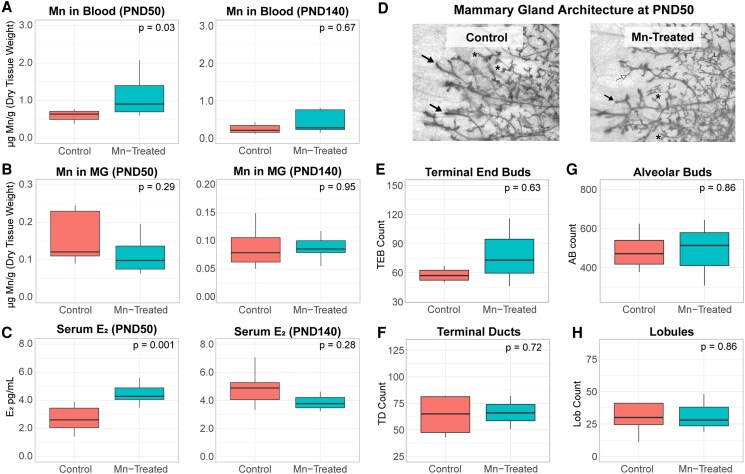
Post-VO Mn exposure intermittently increases endogenous E_2_ but does not alter mammary gland development. Boxplots showing concentration of Mn in blood (A) and mammary glands (B) from control and Mn-treated females at PND50 (left) and PND140 (right) following post-VO exposure (C). Boxplots displaying serum estradiol levels in control and Mn-treated females at PND50 (left) and PND140 (right) following post-VO exposure (D). Representative mammary gland whole mounts from control and Mn-treated females and respective quantification of terminal end buds (E), terminal ducts (F), alveolar buds (G), and type I lobules (H). All boxplots display the median, interquartile range, and minimum and maximum values for each group. *P* values determined using Wilcoxon rank-sum exact tests. AB, alveolar bud; E_2_, estradiol; L, lobules; Lob, lobule type 1; PND, postnatal day; TD, terminal duct; TEB, terminal end bud. Black arrows: TEBS; white arrows: TDs; asterisks: ABs.

Our MnPP model is predicated on the dosing protocol, in that prepubertal Mn exposure results in central precocious puberty, increased mammary epithelial ERα expression, and accelerated mammary gland development (26, 38). To determine if those noted changes are restricted to a specific developmental window or solely due to Mn, we exposed females to Mn after pubertal onset (ie, VO; Supplemental Figure 1 (29)). Post-VO exposure to MnCl_2_ for 18 days increased the mean concentration of Mn found in blood at PND 50 compared to controls (Fig. 7A, left; Supplemental Table 3 (29)). By PND 140, 90 days after the last Mn dose, mean Mn levels in blood from treated females were similar to controls (Fig. 7A, right; Supplemental Table 3 (29)). In addition, we confirmed that Mn did not accumulate in mammary glands at either time point, regardless of treatment status (Fig. 7B, Supplemental Table 3 (29)). Post-VO Mn supplementation did result in increased circulating levels of E_2_ at PND 50 (Fig. 7C, left); however, by PND 140, there was no difference in serum E_2_ levels between Mn-treated females and controls (Fig. 7C, right).

Regardless of the intermittent spike of E_2_ and elevated blood Mn levels at PND 50, post-VO administration of Mn did not appear to alter mammary growth, as indicated by analysis of mammary gland ductal architecture. Whole mount analysis revealed no difference in the number of differentiated ductal structures in mammary glands from control and Mn-treated female rats at PND 50 (Fig. 7D-7H). Specifically, the mean number of terminal end buds (Fig. 7E), terminal ducts (Fig. 7F), alveolar buds (Fig. 7G), and lobular type 1 (Fig. 7H) were unchanged between groups. Furthermore, a luciferase reporter assay determined that a range of MnCl_2_ doses within human intake-range (Supplemental Table 1 (29)) could not directly activate canonical ER signaling in human MCF7 breast cancer cells (Supplemental Figure 2 (29)). There were also no differences in the mean concentration of Mn found in key estrogen-responsive tissues (uterus, ovaries, and hypothalamic/preoptic areas) of treated females at either time point compared to controls (Supplemental Table 3 (29)). Last, post-VO exposure to Mn had no effect on estrous cycle length (mean length in days ± SD: control: 4.7 ± 0.39; Mn-treated: 4.9 ± 0.37), number of cycles during dosing (mean no. cycles ± SD: control: 3.5 ± 0.41; Mn-treated: 3.5 ± 0.37), or length of estrus (mean days of estrus ± SD: control: 1.6 ± 0.22; Mn-treated: 1.5 ± 0.24). Overall, post-VO Mn supplementation had no lasting phenotypic effect on the outcomes we evaluated relevant to the hypothalamic-pituitary-gonadal axis or the adult mammary gland.

## Discussion

Multiple epidemiological studies have implicated early or precocious puberty as a breast cancer risk factor (1, 3, 4), but no study to date has provided a mechanistic explanation for this relationship. This is important because the average age of pubertal onset in girls has consistently decreased over the past several decades, with 95% of central precious puberty cases being idiopathic (39). Further, the COVID-19 pandemic resulted in a spike of precocious puberty cases (40), renewing concerns of associated breast cancer. These concerns are especially pertinent with regard to health equity, as pubertal timing disparities have been reported, with Black girls simultaneously experiencing earlier pubertal onset and higher burden of breast cancer mortality than non-Hispanic White girls (41, 42). In recent years, our group has characterized an intriguing model of central precocious puberty in female rats, identifying Mn as a natural activator of the pubertal process (38). Specifically, early-life Mn supplementation results in accumulation of the element in the hypothalamus where it stimulates release of the hormonal milieu that centrally controls pubertal onset (eg, kisspeptin, GnRH), resulting in ovarian secretion of E_2_ (26). Using this model, we recently demonstrated that MnPP, through an earlier rise in serum E_2_, induces precocious mammary gland development resulting in chronic proliferation and ductal hyperplasia in the adult gland (26). Here, we utilized our MnPP model to investigate this relationship. Our findings suggest that precocious puberty alters hormonal regulation of proliferation in the prepubertal mammary gland, resulting in an autocrine-mediated proliferative phenotype that is commonly observed in human benign breast disease and breast cancer (14, 18-20). We further highlight puberty as a critical developmental window of susceptibility, demonstrating that the timing of E_2_ exposure may be of more importance in precocious puberty-related breast cancer risk than the exposure alone.

Prior to ovulation, growth of the prepubertal mammary gland is primarily hormone independent, despite the expression of ER and PR in the gland (43). As ovulation approaches (ie, onset of puberty), E_2_ becomes the primary proliferative stimulus in the mammary gland, inducing ER transcriptional activity and dictating development primarily through paracrine-mediated proliferation (15, 17). In line with this, we demonstrated that only 2% of total mammary epithelial cells coexpress both PR and PCNA in normally developed prepubertal female rats. This frequency is consistent with a previous study reporting that 2.6% of mammary epithelial cells in young 45 day old female rats coexpressed PR and the proliferative marker, BrdU (44). By young adulthood (PND 120), we saw the population of PR+/PCNA+ cells had fallen to 0.99%, which is similar to what has been reported in several studies utilizing single-cell sequencing from adult human normal breast (45, 46). Thus, the proliferating HR+ cell population is very small in the normal prepubertal mammary gland and appears to remain this way in the young, normally developed postpubertal and adult gland.

A characteristic of premalignant hyperplasia and breast cancer is an increase in the percentage of proliferating ER+/PR+ cells, where hormone-regulated proliferation is primarily autocrine-mediated and directly correlates with breast cancer risk (14, 18-20, 44). Thus, modification of this cell population may have important implications for breast cancer prevention. Supporting this theory, pregnancy, a major reproductive event associated with decreased breast cancer risk, has been shown to decrease the prevalence of proliferating HR+ cells (44). Our data extend this previous work to highlight puberty as another major reproductive event where this population of cells is modified. Herein, when mammary gland development was precociously induced in Mn-treated females, the percentage of proliferating HR+ cells increased significantly relative to normally developed females. We therefore suggest that early puberty may alter the normal balance of epithelial cell types in the developing mammary epithelial landscape, thereby changing the way the gland responds to its surrounding environment and potentially altering susceptibility to later life disease.

It is intriguing that earlier-than-normal pubertal onset may alter hormonal influence of proliferation in the mammary gland. Histological and molecular changes observed in the mammary glands of MnPP females coincided with a 2-fold increase in serum E_2_ (26), suggesting that these changes, at least in part, are hormone induced. We observed a significant increase in FOXA1 protein expression in MnPP females at PND 30, an ER co-regulator essential for hormone responsiveness in both normal mammary gland development and breast cancer (47). Importantly, increased FOXA1 expression has been shown to augment ER transcriptional activity, consequently amplifying normal ER function (10). Given this, we observed a significant increase in expression of ER target proteins, c-Myc and AREG, both of which play important roles in normal mammary gland development and breast cancer when aberrantly expressed (48, 49). Of special interest, AREG is a vital paracrine factor secreted from HR+ cells to induce proliferation in neighboring HR- cells in direct response to E_2_ stimulus (48). Although the proliferative actions of AREG are primarily paracrine in fashion, AREG has also been hypothesized to act in an autocrine manner to stimulate proliferation of HR+ cells (18, 50, 51). These data complement our previous work showing that Mn-induced precocious mammary gland development correlates with a significant increase in proliferation and phosphorylation of ERK½ (26), a response consistently seen following AREG induced proliferation (48). Furthermore, these findings suggest that the overexpression of AREG observed in precociously developed mammary glands could account, at least in part, for the increased percentage of proliferating HR+ mammary epithelial cells observed in MnPP females at PND 30, ultimately resulting in an altered mammary epithelial landscape.

Consistent with our previous reports, the robust proliferation observed in prepubertal mammary glands of MnPP females was sustained at PND 120, whereas proliferation had naturally slowed in control glands. Previously, we showed that this sustained proliferation resulted in ductal hyperplasia in 20% of Mn-treated females, in addition to histological observations of reactive stroma, disorganized luminal epithelial growth, and ductal filling (26). Clinically, a high percentage of human hyperplasia lesions are HR+ (52). In this regard, histological analysis revealed a significant increase in the percentage of both ERα and PR expressing mammary epithelial cells in precociously developed mammary glands compared to controls at PND 120, a phenotype clinically seen in human ER+ breast cancer (53), and that has been observed in histologically normal tissue adjacent to invasive breast lesions (19). In addition to cell proliferation, E_2_ has also been shown to increase PR synthesis in normal human breast (14). We observed a significant increase in protein expression of PR-B at PND 120. Supporting our proliferative phenotype, P_4_ has been shown to induce proliferation in the mammary gland via PR-B either directly or via secretion of paracrine mediators (53). We observed a significant increase in protein expression of one such factor, EREG, which is primarily stimulated by P_4_ (48) and to a lesser extent by E_2_ (18). Interestingly, the overexpression of both AREG and c-Myc observed at PND 30 was not significant at PND 120, suggesting that P_4_ was the primary stimulus of paracrine proliferation at this time point via stimulation of EREG. This was not surprising given that in the normal adult mammary gland, P_4_ is the primary proliferative stimulus, producing the highest levels of proliferation in the breast during the luteal phase of the menstrual cycle, when P_4_ is highest (54-56). Supporting the possibility of a facilitating role of P_4_ in the hyperplasic phenotype of precociously developed adult mammary glands, we observed a significant increase in expression of Sp1 in mammary glands of Mn-treated females at PND 120. Sp1 is required by PR for transcriptional activation of multiple target genes including cell cycle regulators (57) and is a marker of poor prognosis in breast cancer (58). It is important to note that although Sp1 is necessary for many PR downstream effects, it has also been shown to mediate ERα transcriptional activity (59). Thus, our data suggest that the previously observed hyperplasic phenotype of precociously developed adult mammary glands is due, in part, to increases proliferation following aberrant expression of both ERα and PR.

Our results also support previous studies illustrating the relationship between mammary epithelial proliferation and age (44, 19, 60). In line with the less proliferative nature typical of virgin adult mammary glands relative to younger pubertal glands (13), we observed a trending, though nonsignificant, decrease in the percentage of proliferating cells in control mammary glands from PND 30 to PND 120. Despite this trending reduction, approximately half of all remaining proliferating cells in the adult virgin gland were also ER+, whereas approximately one tenth were PR+. These data support that the normal population of proliferating ER+ cells increases with chronological age, as has been shown in the aged human breast, despite an overall reduction in proliferation (19, 60). However, to our knowledge, this population of cells has not previously been characterized with regard to precocious puberty—an event that may be associated with advanced biological age (61). Herein, MnPP adult mammary glands were significantly more proliferative than controls at PND 120 and displayed a significant increase in the total percentage of proliferating ER+ epithelial cells and proliferating PR+ epithelial cells. Furthermore, 79.34% of all proliferative cells were also ER+ and 19.79% were also PR+, suggesting that this increase was not simply due to an overall increase in proliferation, but to a change in the primary mechanism of hormone-regulated proliferation (ie, autocrine vs paracrine). Future studies are needed to investigate the relationship between puberty-associated proliferative mechanisms and breast cancer risk. Further, our MnPP model may provide utility in investigating the relationship between precocious puberty and biological vs chronological breast tissue age in the context of cancer risk (62).

Although further studies will be required to elucidate a mechanism explaining the shift in hormone-regulated proliferation, given the tight association between ER and PR with the cell cycle (57), these changes could be attributed to deregulation of cell-cycle control. Supporting this, we previously showed that MnPP resulted in increased protein expression of AP2α and p53 in PND 120 glands, an association known to mitigate the ability of p53 to adequately regulate the cell cycle (63). Sp1, inducible by both ER and PR and upregulated in MnPP glands at PND120, also has known associations with p53 and has been shown to counteract its inhibitory actions (64). Mutations in TP53 are the most frequent alteration in breast cancer and are accepted as an early step in human breast carcinogenesis (65). Interestingly, TP53 mutations are enriched in breast tumors from Black women relative to non-Black women relative to non-Black women (42, 66), a high-risk patient population with the lowest median age of pubertal onset, and where earlier puberty is associated with ER-negative breast cancer (41). In line with this, basal-like and HER2-enriched breast cancer subtypes, which are often ER-negative and harbor the highest burden of TP53 mutations, are hypothesized to arise from early luminal progenitor cells (67, 68). We also observed a significant decrease in expression of cell-cycle inhibitor p27 in luminal epithelial cells from MnPP females at PND 120. p27 has been suggested to block autocrine proliferation in PR-B expressing cells (55), whereas loss of p27 in PR+ breast cancer is associated with bypassing of normal cell-cycle controls (69). Collectively, these data support a role of hormone-induced cell-cycle deregulation in the mammary gland of precociously developed female rats, with important implications for breast cancer racial disparities. Future studies are needed to determine whether the histological and molecular changes we observed among pubertal luminal epithelial cells reflect luminal progenitors.

It has been hypothesized that earlier pubertal onset may increase breast cancer risk by increasing lifetime exposure to estrogen through additional menstrual cycles; however, this has been a difficult relationship to investigate due to lack of a strong experimental model. As such, a strength of our study was the use of our MnPP model, where Mn supplementation naturally and centrally activates the pubertal process without accumulation in the mammary gland and allows for investigation of puberty and breast cancer risk without the use of steroidogenic compounds or diets that can directly impact breast development or the surrounding microenvironment (70-72). However, given a role of Mn in the maintenance of immune responses (73) and the growing understanding of the immune microenvironment in normal breast function, our current study does not rule out systemic impacts on nonepithelial components of the mammary gland following Mn supplementation, which may contribute to changes in the gland. Another strength of our study was the ability to assess post-VO Mn exposure. We showed that a Mn-induced post-VO rise in serum E_2_ had no effect on mammary gland development, as determined from analysis of differentiated ductal structures. Furthermore, we determined that Mn does not possess estrogenic properties and have confirmed in both our previous (26) and current work that Mn does not accumulate in the pre- or post-VO rodent mammary gland. As such, our results provide evidence to support puberty as a critical development window of susceptibility in breast cancer risk, with sustained changes from an early rise in serum E_2_ and not just E_2_ exposure in general. Our analysis also had limitations. Because of challenges with tissue availability, we were unable to assess colocalization of HRs and proliferative markers in the post-VO gland, nor the colocalization of ER and PCNA at PND 30. However, our previous work characterized ER and related signaling proteins at PND 30 (26). Nevertheless, although most etiologic studies of breast cancer emphasize ER, our molecular analysis allowed for analysis of both ER- and PR-associated targets across 2 developmental time points, elucidating important insights for both clinically utilized breast cancer biomarkers.

**Figure 8.**
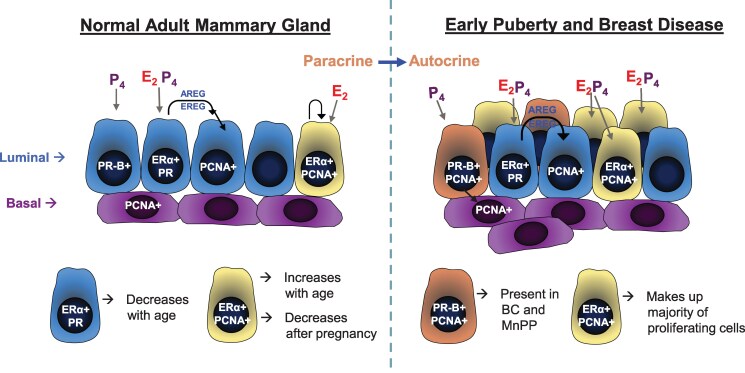
Proposed steroid-driven proliferation in normal adult mammary gland compared to early puberty and breast disease.

In conclusion, our findings fill a research gap to further understand precocious puberty as a breast cancer risk factor. Using our novel MnPP model, we clearly demonstrate that precocious puberty alters hormonal regulation of proliferation in the prepubertal mammary gland by augmenting ER- and PR-regulated proliferative pathways, resulting in an altered mammary gland epithelial landscape that may be more sensitive to cycling E_2_ and P_4_ (Fig. 8). These pubertal changes persist in the adult mammary gland, resulting in a hormone-induced predominance of autocrine-regulated cellular proliferation and premalignant transformation. These findings will have important implications for informing precision prevention strategies in breast cancer and promoting equitable health outcomes.

## Data Availability

Some or all datasets generated during and/or analyzed during the current study are not publicly available but are available from the corresponding author on reasonable request.

## Abbreviations

AREG: amphiregulin
E_2_: estradiol
ER: estrogen receptor
EREG: epiregulin
EtOH: ethanol
HR: hormone receptor
IF: immunofluorescence
IHC: immunohistochemistry
Mn: manganese
MnPP: manganese-induced precocious puberty
P_4_: progesterone
PND: postnatal day
PR: progesterone receptor
RT: room temperature
VO: vaginal opening
